# Four-Layer Surface Plasmon Resonance Structures with Amorphous As_2_S_3_ Chalcogenide Films: A Review

**DOI:** 10.3390/ma16186110

**Published:** 2023-09-07

**Authors:** Aurelian Popescu, Dan Savastru, Mihai Stafe, Nicolae Puscas

**Affiliations:** 1 National Institute of R & D for Optoelectronics INOE 2000, 409 Atomistilor Str., 077125 Magurele, Romania; dsavas@inoe.ro; 2 Department of Physics, University Politehnica of Bucharest, 313 Splaiul Independentei, 060042 Bucharest, Romania; mihai.stafe@upb.ro (M.S.); nicolae.puscas@upb.ro (N.P.)

**Keywords:** surface plasmon resonance, optical sensors, amorphous chalcogenide materials, optical hysteresis

## Abstract

The paper is a review of surface plasmon resonance (SPR) structures containing amorphous chalcogenide (ChG) films as plasmonic waveguides. The calculation method and specific characteristics obtained for four-layer SPR structures containing films made of amorphous As_2_S_3_ and As_2_Se_3_ are presented. The paper is mainly based on our previously obtained and published scattered results, to which a generalized point of view was applied. In our analysis, we demonstrate that, through proper choice of the SPR structure layer parameters, we can control the resonance angle, the sharpness of the SPR resonance curve, the penetration depth, and the sensitivity to changes in the refractive index of the analyte. These results are obtained by operating with the thickness of the ChG film and the parameters of the coupling prism. Aspects regarding the realization of the coupling prism are discussed. Two distinct cases are analyzed: first, when the prism is made of material with a refractive index higher than that of the waveguide material; second, when the prism is made of material with a lower refractive index. We demonstrated experimentally that the change in reflectance self-induced by the modification in As_2_S_3_ refractive index exhibits a hysteresis loop. We present specific results regarding the identification of alcohols, hydrocarbons, and the marker of *E. coli* bacteria.

## 1. Introduction

Plasmonics is a research field that explores the confinement of the electromagnetic field over dimensions of the order of the wavelength. The basic phenomenon responsible for spatial subwavelength confinement of the electromagnetic field is the interaction between the electromagnetic radiation and the conduction electrons of the metallic interfaces or metallic nanostructures, leading to an enhanced optical near field.

The dimensions of the photonic devices are restricted by the diffraction limit, which is of the order of half the wavelength for conventional optical devices. Accordingly, the diffraction limit is of the order of 250 nm in the visible domain, which is approximately an order of magnitude larger than the electronic component’s dimension (~10 nm). To overcome the diffraction limit, metal-insulator structures were developed, which may confine the light near the interface at dimensions shorter than the optical wavelength due to the surface plasmons (which represent waves of the electric charge density at the metal–insulator interface) [1]. However, the light attenuation in plasmonic structures is large, so the propagation distance is of the order of mm due to the forced oscillations of the free electrons under the electromagnetic field.

The plasmon propagation can be used for signal routing along the conductive nanowires. Since the surface plasmons do not involve electric charge displacement, the effects of inductance and capacity (which reduce the performance of the integrated circuits) do not occur. There are many specialized monographs in the literature [2,3,4,5,6] on problems linked to plasmonics.

The proposal of Kretschmann to use prism coupling [7] very soon led to the development of plasmonic sensors [8,9,10], the results being particularly impressive for biological sensors [11,12,13], which are selective to analytes. Other interesting types of sensors were described in [14,15].

Fundamentals of the plasmons in multilayer structures can be found in Maier’s book [16]. Davis [17] proposed the matrix method for the calculation of light interaction with multilayer structures made of metals and insulators. Other authors [18,19,20] used the matrix method to determine the resonance characteristics. Economou [21] and Burke et al. [22] derived the dispersion relation. This was analyzed for different multilayer configurations, but the solutions were obtained for special symmetry structures only. Opolski [23] performed numerical simulations of the plasmon resonances in planar structures. However, the matrix method does not enable the calculation of the electromagnetic field distribution in the structure layers.

Chalcogenide (ChG) materials have high transparency in the IR region as well as a high refractive index [24]. A surface plasmon resonance (SPR)-based biosensor using the amorphous ChG material Ge_20_Ga_5_Sb_10_S_65_ (called 2S2G) as a coupling prism [25] enabled a sensing limit for refractive index variation of 5 × 10^−5^.

Chalcogenide materials offer the possibility of improving the characteristics of conventional SPR sensors. Thus, in [26], a chalcogenide glass-based sensor applicable in the IR region was studied. The use of Al film for the chalcogenide glass sensor increased the intrinsic sensitivity by almost 400% as compared with an Au film-based sensor, the material most commonly used in the visible region. The design of IR sensors is an actual task due to the existence of gas absorption lines in the IR spectral domain.

In [25], numerical simulations were carried out to investigate the potentialities of sulfide glass systems as coupling prism materials. An SPR biosensor was set up by using angular interrogation. The calculations performed showed that the detection limit of the sensor was 3 × 10^−5^ RIU. The development of SPR sensors with a chalcogenide thin-film layer was proposed in [27] for bio-applications. The film, accompanied by a graphene layer, demonstrated selective adsorption. Recent studies established plasmon-enhanced photo-stimulated diffusion of silver into GeSe_2_-based chalcogenide thin films [28]. Another study [29] demonstrated a significant increase in the diffraction grating characteristics formed on an inorganic ChG photoresist due to surface plasmons.

Amorphous ChG manifests new characteristics when configured as a metamaterial. In [30], the authors experimentally demonstrated that a nanostructured chalcogenide glass can efficiently generate third-harmonic radiation, leading to a strong UV light source at the nanoscale due to phase locking, despite ordinarily high optical absorption in this region. Later, the authors [31] demonstrated a two-order-of-magnitude improvement of third harmonics in stacked three-layer chalcogenide metasurfaces with respect to a single layer.

Theoretical calculations [32] for permittivity were performed using the Maxwell–Garnett model for composite materials containing metal (Ag, Al) nanoparticles in a dielectric medium. This method paved the way for the realization of new metamaterials, i.e., materials with a negative refractive index. This does not require a periodic distribution of particles, making it very technologically applicable. It was shown that the permittivity can be engineered by changing the inclusion coefficient, which was too small (around 0.05). ChG materials are very promising as host materials because they provide low optical losses.

Another SPR experiment was presented in [33], where the photoinduced modifications in amorphous As_2_Se_3_ [34] were detected due to plasmonic resonance enhancement. Light-to-light modulation was demonstrated in an SPR configuration using ChG materials as the active medium and a rutile coupling prism [35]. As the authors mentioned, the SPR resonance dip was only observed for p-polarized light. We expect that more complex resonance phenomena will occur when thin ChG films with a high refractive index are used as the sensing medium in multilayer SRP configurations.

This review analyzes SPR in multilayer structures, with a focus on four-layer structures that act as planar waveguides. Currently, several SPR-based tools are available on the market. All of them, whether chemical or biological, are based on the three-layer Kretschmann configuration [7]. Four-layer structures containing amorphous ChG materials open up new possibilities in terms of manipulation with the degree of confinement, sensitivity to refractive index changes, and depth of field. Minimization of the optical loss of the structure is achieved by proper selection of the ChG film material and metal film thickness, accounting for the working laser wavelength. The increased refractive index of the ChG film is essential to achieving better field confinement near the surface. Films composed of As_2_S_3_ or As_2_Se_3_ are considered reference materials. They have a high refractive index (2.45–3.0), low optical losses in the transparency band, and can be easily obtained on large metal or dielectric surfaces by vacuum deposition techniques.

The opportunity for chalcogenide photonics was traced in the review of Eggleton and co-authors [36,37]. The papers summarized progress in photonic devices that exploit the optical properties of chalcogenide glasses. They identified the most promising areas, such as mid-infrared sensing, integrated optics, and ultrahigh-bandwidth signal processing.

More recently, the authors [38] have written a review analyzing the concept of new lab-on-chip devices that exploit acousto-optic interactions to create lasers, amplifiers, and other photonics devices. The chalcogenide materials are characterized by a record-high acousto-optic coefficient. Just recently, the authors of [39] presented the road map for emerging photonic technology platforms. Chalcogenide semiconductors play a critical role in this architecture. The first demonstration of on-chip devices using an As_2_S_3_ planar waveguide was presented.

The physical bases related to the interaction of light with plasmon polaritonic waves are elucidated briefly in Section 2. Section 3 presents the transfer matrix method used to calculate resonance curves in multilayer planar structures, particularly the four-layer structure. The results with specific calculations are presented in Section 4. Section 5 presents numerical simulations using the characteristic equation or complete solver to calculate the field distribution in a four-layer structure, with some discussions on the topic of plasmonic resonance. Nonlinear effects in the structure of SPR containing a film of amorphous ChG compounds (As_2_S_3_, As_2_Se_3_) are presented in Section 6. The photo-induced phenomena known in these materials are amplified by the phenomenon of resonance, which promises some applications in active photonic devices. Finally, in Section 7, the main properties of the four-layer structure are presented, which concern the sensor’s applications (Section 7.1 for alcohol identification and Section 7.2 for *E. coli* detection). Discussions and conclusions are given in Section 8 and Section 9.

## 2. Surface Plasmon Resonance (SPR) in a Three-Layer Configuration: Basic Features

The SPR configuration employs the total internal reflection of the light at the prism base. The evanescent field extends through the thin metal film deposited on the base, and it couples with the plasmon polariton wave created at the external metal interface of the metal film. The resonant coupling of the light occurs when the phase velocity of the light parallel to the surface is equal to the velocity of the plasmon, so that surface plasmon polariton (SPP) waves can propagate along the interface between the conductor and dielectric.

The Maxwell equations describing the SPP propagation reduce to a system of coupled Helmholtz wave equations when considering harmonic oscillations, one per layer of the structure. They can be solved for different geometries [16,40,41]. In the case when the planar structure lies in the *xy* plane, which designates the interface of layers, the equations correspond to the one-dimensional case. Every Helmholtz equation is still vectorial and, in the general case of an arbitrary polarization of the incident light, is a system of several equations. The number of equations corresponds to the number of layers. The introduction of the concepts of TM and TE mode allows the reduction of the system to a scalar equation. Finally, the following expression for the propagation constant was obtained [16]:

(1) $$\beta =k_{0}\sqrt{\frac{\varepsilon_{1}\varepsilon_{2}}{\varepsilon_{1}+\varepsilon_{2}}}$$

The conditions required for SPP wave coupling at the metal–dielectric interface are as follows [40]:

(a) Only TM polarization can trigger SPP waves. For this TM polarization, the electric field triggers the oscillation of free electrons due to the electric field component perpendicular to the dielectric–metal interface.
(b) The real permittivity of the metal (*ε*_1*r*_) and dielectric (*ε*_2_) are of the opposite sign. They must satisfy the condition: Re {*ε*_1*r*_} < −*ε*_2_.

The electromagnetic fields decrease exponentially with distance from both interfaces. The depth of penetration is on the order of 250–300 nm in dielectrics (half of the approximate wavelength). In metals, the field penetration is on the order of 10–15 nm due to high absorption. So, a coupled state of plasmon polariton and electromagnetic waves propagate along the metal–dielectric interface.

The optical constants of metals and their dispersion play an important role in ensuring the conditions for the propagation constant to be real. The metal–dielectric interface supports SPP waves only for such metals that have a large enough value of the dielectric constant. The Drude plasma model was formulated in order to calculate the dielectric constant. In this model, the macroscopic polarization leads to the following dispersion relation for permittivity:

(2) $$\varepsilon \left(\omega \right)=1-\frac{\omega_{p}^{2}}{\omega^{2}+i\gamma \omega }$$

Here, $\omega_{p}$ is the plasma frequency. The relative permittivity is complex *ε(ω) = ε_r_(ω) + i ε_r_ (ω)*, where *ε_r_* is the real and *ε_r_* is the imaginary component. For large light frequencies (i.e., *ω ≫ γ*, but *ω < ω_p_*), the relative permittivity *ε(ω)* given by Equation (2) becomes real and negative:

(3) $$\varepsilon \left(\omega \right)=1-\frac{\omega_{p}^{2}}{\omega^{2}}$$

The relative permittivity is generally calculated from the optical constants *n* and *k* as *ε(ω) = (n − ik)*^2^. Tabulated values for the optical constants are given in Palik’s Handbooks [42,43]. Examples of optical constants for usual metals employed in plasmonic experiments were obtained by extrapolation of Palik’s data to the used wavelengths (see Table 1).

From these data, the dielectric constants, which are complex, can be calculated. Aluminum has higher values of the extinction coefficient, which leads to stronger attenuations of SPP waves. The low value of the extinction coefficient is the reason for choosing noble metals such as gold or silver for building plasmonic structures.

**Figure 1:**
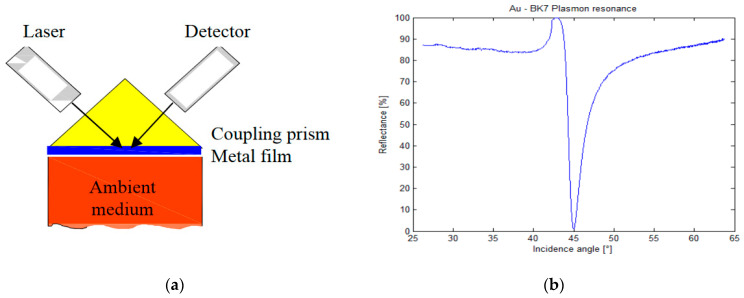
Three-layer SPR configuration (**a**) and the resonance curve obtained experimentally with an Au thin film of 50 nm thickness (**b**).

The analysis concept presented above leads to the formula for the plasmon propagation constant *β*. To excite the SPP waves, the phase-matching conditions for energy and momentum must be fulfilled. Since the propagation constant *β* is greater than the wave vector *k* of the light in the dielectric, the realization of phase-matching conditions is not a trivial problem. Kretschmann and Raether [7] demonstrated that phase-matching conditions can be achieved by using a thin metal film in a three-layer configuration. In this configuration, the top semi-infinite medium is made of a dielectric with a refractive index higher than the bottom one, and the SPP wave can be excited at the bottom metal interface via evanescent waves. Figure 1a presents a three-layer structure with BK7 glass with a refractive index of 1.51 (the coupling prism) at the top and air with a refractive index close to unity or water solutions with a refractive index close to 1.33 (the ambient medium) at the bottom of the metallic film.

The resonance conditions require that the propagation constant *β* (Equation (1)) be equal to the propagation constant of light tangential to the surface:

(4) $$k_{0}n\sin\theta =\beta =k_{0}\sqrt{\frac{\varepsilon_{1}\varepsilon_{2}}{\varepsilon_{1}+\varepsilon_{2}}}$$

The incidence angle θ is measured from the normal to the interface. From the experimental curve, which corresponds very well to the calculated one, we can see in Figure 1b that the angle incident to the SPR interface is near 45°.

Due to refraction restrictions, this resonance angle can be obtained when the light beam is directed normally to an optical prism with a 90-degree angle. Small adjustments are made by fine rotations of the table since the resonance angle is very sharp, on the order of tenths of a degree.

The Formula (4) for the propagation constant assumes the metal film to be thick. When the film thickness decreases, the SPP’s modes will couple to each other, producing a shift in the resonance angle that was first calculated by Kretschmann [44]. The shift of the resonance curve was still small when the film thickness was ~50 nm. This one corresponds to a drop in reflectance near zero for probe light of 633 nm wavelength.

In the book of Sophocles [45], the solutions for three-layer plasmonic waveguide structures are presented. The analytical expression for the reflectivity represents the Airy formula for the three-layer structure [46]. Abeles’s 2 × 2 matrix approach [47] may be employed for the calculation of the reflectivity of a multilayer structure.

## 3. Transfer Matrix Method for Calculating the SPR Resonance Curves in Multilayer Configurations

For multilayer configurations, it is not possible to obtain an explicit solution for the reflectivity. The reflectivity can be obtained for the general *N*-layer case in terms of characteristic transfer matrices [47]:

(5) $$[\begin{array}{c} U_{1}\\ V_{1} \end{array}]=M_{2}\cdot M_{3}\cdots M_{N-1}\cdot [\begin{array}{c} U_{N-1}\\ V_{N-1} \end{array}]$$

Calculations may be performed for both *p* and *s* polarizations. The following notations can be used:

(6) $$q_{pj}=\frac{\sqrt{e_{pj}-{\left(n_{p1}\sin\theta \right)}^{2}}}{e_{pj}}\;q_{sj}=\sqrt{e_{sj}-{\left(n_{s1}\sin\theta \right)}^{2}}$$

(7) $$\beta_{pj}=\frac{2\pi d_{j}}{\lambda }\sqrt{e_{pj}-{\left(n_{p1}\sin\theta \right)}^{2}}\;\beta_{sj}=\frac{2\pi d_{j}}{\lambda }\sqrt{e_{sj}-{\left(n_{s1}\sin\theta \right)}^{2}}$$

In the above notations, *s* and *p* designate the polarization, while *j* is the layer’s number. For each layer number *j*, the transfer matrices *M_pj_* and *M_sj_* are calculated:

(8) $$M_{pj}=\left(\begin{array}{c} \cos\beta_{pj}\;\;\;\;\;\;\;\;\;\;\;\;\;\;\;\;\;\;-i\frac{\sin\beta_{pj}}{q_{pj}}\\ -i\;q_{pj}\sin\beta_{pj}\;\;\;\;\;\;\;\;\;\;\;\;\cos\beta_{pj}\;\; \end{array}\right)\;M_{sj}=\left(\begin{array}{c} \cos\beta_{sj}\;\;\;\;\;\;\;\;\;\;\;\;\;\;\;\;\;\;-i\frac{\sin\beta_{sj}}{q_{sj}}\\ -i\;q_{sj}\sin\beta_{sj}\;\;\;\;\;\;\;\;\;\;\;\;\cos\beta_{sj}\;\; \end{array}\right)$$

Next, the total transfer matrices *T_p_* and *T_s_* are calculated:

(9) $$T_{p}=\prod_{j=0}^{N}M_{pj},\;T_{s}=\prod_{j=0}^{N}M_{sj}$$

Finally, the reflectance *R_p_* and *R_s_* are calculated:

(10) $$R_{p}=\left|\frac{\left(T_{p}\left(0,1\right)q_{pN}+T_{P}\left(0,0\right)\right)q_{p1}-\left(T_{p}\left(1,1\right)q_{pN}+T_{P}\left(1,0\right)\right)}{\left(T_{p}\left(0,1\right)q_{pN}+T_{P}\left(0,0\right)\right)q_{p1}+\left(T_{p}\left(1,1\right)q_{pN}+T_{P}\left(1,0\right)\right)}\right|\;R_{s}=\left|\frac{\left(T_{s}\left(0,1\right)q_{sN}+T_{s}\left(0,0\right)\right)q_{s1}-\left(T_{s}\left(1,1\right)q_{sN}+T_{s}\left(1,0\right)\right)}{\left(T_{s}\left(0,1\right)q_{sN}+T_{s}\left(0,0\right)\right)q_{s1}+\left(T_{s}\left(1,1\right)q_{sN}+T_{s}\left(1,0\right)\right)}\right|$$

SPR computations were realized by using scripts written in MATLAB: the first script enables the calculation of the structure reflectivity as a function of the incidence angle and the determination of the resonance angle. The second script enables us to calculate the structure’s reflectivity as a function of the film’s refractive index.

## 4. Four-Layer SPR Configuration with Amorphous ChG Film: Simulation Results

The plasmonic resonance structure proposed by Kretschmann was developed and found applications as the newest photonic devices. Several companies have realized and commercialized successful optical sensors based on SPR. These conventional schemes basically represent a three-layer configuration: (1) a semi-infinite prism made of oxide glass; (2) a gold film with a thickness of 40–50 nm; and (3) an ambient environment, which represents solutions of various chemicals in water.

**Figure 2:**
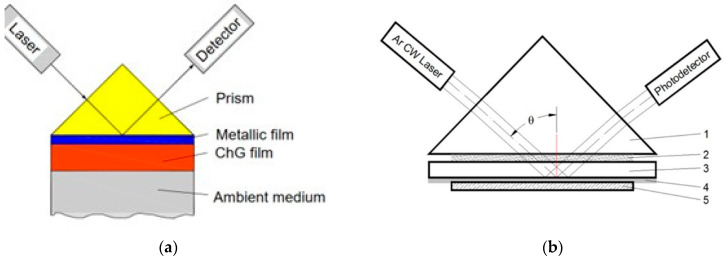
Four-layer SPR configurations with ChG film as a waveguide: (**a**) concept and (**b**) experimental schematic. The setup contains the following: 1—optically transparent prism; 2—optical adhesive or immersion oil; 3—glass substrate; 4—thin gold film; 5—amorphous ChG film.

The properties of a plasmonic resonant structure can change drastically if a thin film of high refractive index is deposited on the metallic film. The structure is transformed into a four-layer configuration, which contains a transparent dielectric film as a waveguide (Figure 2a).

The refractive index and film thickness are parameters that ultimately determine the sensitivity of sensors. Amorphous ChG materials are a good candidate for this purpose because they can be deposited on a wide range of substrates. In addition, the nonlinear effects and the photoinduced change in refractive index known in these materials may lead to the development of new photonic devices. The incorporation of these materials in resonant structures leads to considerable amplification of the effects of interaction with light.

In Figure 2b, the substrate (3) with the deposited gold film (4) and the ChG film (5) constitute a planar plasmonic chipset. In photonic devices the chipset is attached to the prism permanently by an adhesive. During the use of these structures as sensors, the surface with the deposited thin films often deteriorates. In this case, the chipset is attached to the prism base using immersion oil. The chipset can be changed, but the prism remains the same.

**Figure 3:**
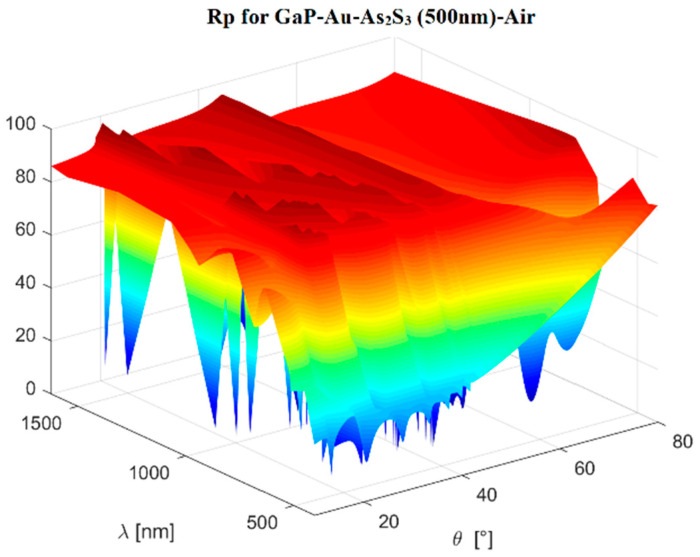
A 3D representation of the reflection coefficient of p-polarized light for SPR structures with 40 nm thick Au films and 500 nm thick As_2_S_3_ films. More intense red colors mean higher field intensity. The blue color means that the value of the field is close to zero. For a given wavelength *λ*, multiple resonant angles *θ* are possible due to the high refractive index of the GaP prism. The resonance angle corresponds to the dip in reflectivity. As shown in the figure, resonant angles can be in a wide range, from 20° to 60°. The resonance curve is sharper for angles close to 20°, and a narrower resonance means a higher quality factor.

Below, we describe the calculations and analysis of the results obtained based on the method presented above. The influence of various parameters of materials on the characteristics of the SPR structure was analyzed. Simulation considered the thickness variation of metal and dielectric layers in a four-layer structure: gallium phosphide (material of the coupling prism)–Au (metal layer)–As_2_S_3_ (dielectric layer)–air. We considered three thicknesses of the Au film (i.e., 40, 45, or 50 nm) and four thicknesses of 300, 500, 700, and 1000 nm for As_2_S_3_. The refractive index of GaP *(n* = 3.1) is higher than the As_2_S_3_ refractive index (*n* = 2.45). Figure 3 presents 3D mappings for p-polarized reflectance *Rp*. The calculations are carried out for usual laser sources such as laser diodes or DPSS lasers and for incidence angles *θ* ranging between 10° and 80°.

**Figure 4:**
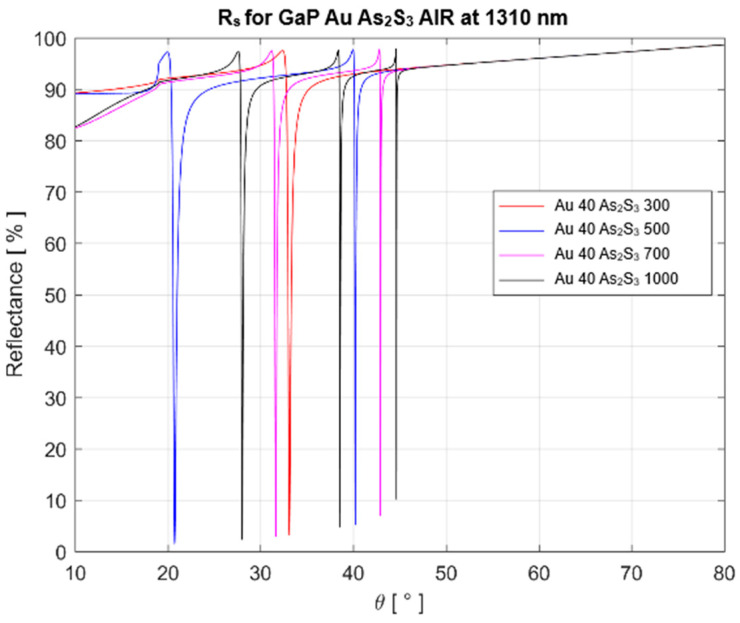
Reflectance as a function of incidence angle θ for *s* polarizations at the wavelength of 1310 nm. The SPR structure has an Au film (40 nm thickness) and an As_2_S_3_ film with different thicknesses in nm. For a thickness of 300 nm, only one resonance at 33° occurs. For larger thicknesses, two sharp resonances can be observed, meaning that higher sensor sensitivities can be obtained.

**Figure 5:**
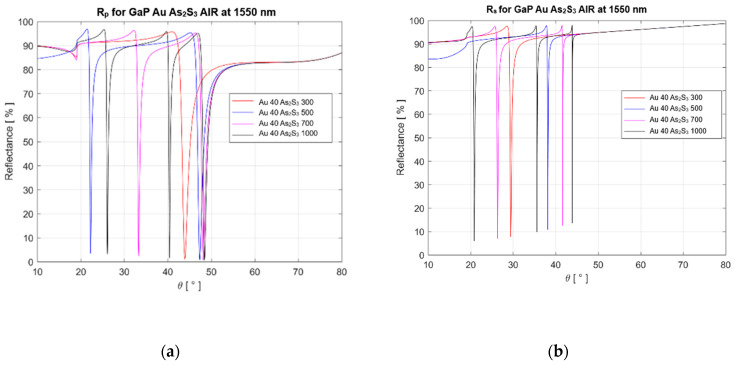
The reflectances R_p_ and R_s_ as a function of incidence angle θ for two polarizations (p and s) at a wavelength of 1550 nm: (**a**) $R_{p}$ for the SPR structure with Au film (40 nm thickness) and As_2_S_3_ films with different thicknesses in nm; (**b**) $R_{s}$ for the SPR structure with Au film (40 nm thickness) and As_2_S_3_ films with different thicknesses in nm. For the same film thickness, the values of the resonance angles corresponding to figure (**b**) are lower for s polarization. The reflections for the resonance angle are closer to zero for p polarization, while for s polarization they are not. At large thicknesses (700 nm and 1000 nm), two resonance angles corresponding to different waveguide modes can be observed.

We carried out calculations for two wavelengths of probe light that are usually employed in optical fiber communications: 1310 nm and 1550 nm. The results are presented in Table 2 and Figure 4 for the 1310 nm wavelength and in Table 3 and Figure 5 for the 1550 nm wavelength.

The transfer matrix approach is an efficient and simple tool for the design of SPR structures. The computer simulations are fast. However, the method does not enable the calculation of the field distribution or the wave attenuation.

## 5. Characteristic Equation Method

For such targeted applications, we have developed the characteristic equation; however, it is unfortunately a transcendental type of equation, which requires more computing time. The characteristic equation must be solved numerically using, for example, MATLAB to find the real and imaginary parts of the propagation constant. The structure parameters working at a laser wavelength of 633 nm are as follows: a BK7 glass prism, a metallic layer of Au (gold), a ChG layer of As_2_S_3_, and lastly, a semi-infinite layer of air. The Au film thickness is 50 nm, and the ChG film thickness *d* may vary between 200 and 1600 nm. The refractive index of the ChG film was considered to be 2.45. The optical constants of Au were taken from the paper by Rakic [48], and the refractive index at 633 nm wavelength is n=0.19−3.25i. The real part of the effective refractive index *N_eff_* = *β*/*k_0_*, where *k*_0_ is the wave vector in air and the propagation constant *β* is a function of ChG film thickness *d*.

**Figure 6:**
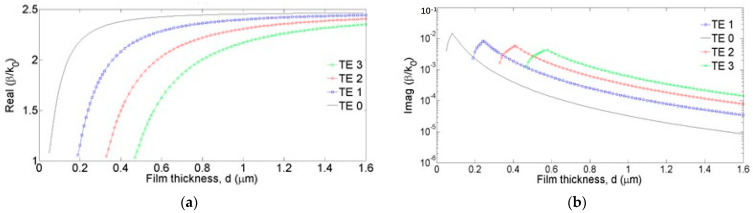
Real part (**a**) and imaginary part (**b**) of the propagation constant for the TE modes.

Some results for the propagation constant *β* are presented in Figure 6 for TE modes.

In resonance conditions, all the energy of light is absorbed by oscillating electrons, which leads to zero intensity in the reflected light. To realize the resonance interaction, the light wave vector component parallel to the interface must be equal to the propagation constant of the SPP wave (*k_0_ n_p_ sin θ*) = *β*, and *β*/*k_0_* = *N_eff_*, where *N_eff_* is the effective refractive index of the propagating wave. The maximum value of *N_eff_* is obtained for *θ* = 90°. We consider that the prism and substrate are made of the same material.

The photon energy can be effectively coupled to SPP mode only when the *N_eff_* is in the range 1.0÷1.5. It means that the plasmonic waveguide mode can only be excited for certain film thicknesses and only one mode at a time. It is an interesting result that the four-layer SPR configuration, which contains a high refractive index film (like amorphous ChG glass), can be coupled with light by using a prism of a lower refractive index (like BK7 glass). The high refractive index gives better light confinement to the surface, meaning better sensitivity in the case of sensors.

**Figure 7:**
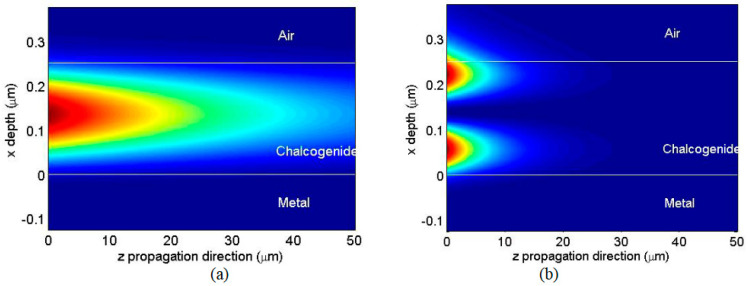
Two-dimensional plot of the intensity distribution in the SPR structure with a ChG film (with 0.25 μm thickness) for the TE_0_ (**a**) and TE_1_ (**b**) modes at 633 nm wavelength. More intense red colors mean greater intensity of the field. The dark blue color means that the field has a near-zero value. For the TE_0_ mode, the propagation of plasmonic waves is mainly through the center of the film, while for the TE_1_ mode, the energy propagates closer to the interface of the chalcogenide film with the adjacent media.

The electric and magnetic field distribution of the TM and TE modes can also be found (Figure 7) [20]. For example, the whole electromagnetic field for the TE modes excited within the SPR structure can be derived by calculating one component of the electric field (e.g., $E_{y}$, which is perpendicular to the plane of incidence) within every layer of the SPR structure. The one-dimensional propagation equation for $E_{y}$ within every layer of the SPR structure is given as [19,20]:

(11) $$\frac{\partial^{2}E_{y}}{\partial x^{2}}+\left(k_{0}^{2}n^{2}-\beta^{2}\right)E_{y}=0$$

where $i=1÷4k_{0}$ is the vacuum wave vector; β, $\beta =k_{0}n_{1}sin(\theta )$ given by Equation (4), is the “axial” wavevector along the *z* axis; and *n* is the refractive index of the layer.

The equation system (11) contains the equations for each medium of the SPR structure, which are bonded by continuity conditions. The general solution of these differential equations is a superposition of progressive and regressive waves along the *x* axis for every region of the SPR structure, except for the last medium (air), where the emerging wave is described as a progressive wave. The continuity equations for the magnetic and electric fields at the three interfaces of the waveguide structure are fulfilled if the magnetic field and its spatial derivative over $n^{2}$ are continuous. The continuity equations for the three interfaces are equivalent to six algebraic equations that enable calculation of the wave’s amplitudes within the SPR layers, considering the amplitude of the incident wave and the propagation constant as input parameters. These equations are solved numerically in MATLAB with ordinary solvers, and the amplitude and power of the wave reflected at the glass–metal interface are calculated relative to the power of the incident wave.

## 6. Nonlinear Behavior of SPR Structures That Contain Amorphous As_2_S_3_ Thin Films

Amorphous solid materials enable physical phenomena that are not observed in crystalline materials. The most important phenomenon for optoelectronics is the modification of the optical constants, either real or imaginary, under laser irradiation. Under low-intensity laser illumination, the optical bandgap decreases in amorphous As_2_S_3_ and As_2_Se_3_. Photodarkening in As_2_S_3_ or As_2_Se_3_ [49] is associated with structural changes. It has a linear dependence of the redshift of the absorption edge on intensity in the domain of 50÷200 mW/cm^2^. Photodarkening in amorphous As_2_S_3_ or As_2_Se_3_ can be improved by doping with rare earth elements, as shown in [50]. The studies were carried out on As_2_Se_3_ nanolayers [51] and demonstrated reliably the oxidation of arsenic during illumination. This process may be implicated in the long-term, hundreds-of-minutes modification of optical transmission in amorphous chalcogenides. More complex photoinduced changes also take place in the nanosecond up to the femtosecond domain [52] when ChGs exhibit transient absorption (TA) triggered by recombination of self-trapped excitons.

The photoinduced changes in optical transmission were first observed experimentally by de Neuville, and many theoretical models were proposed for describing the experimental findings. For example, the models of Street [53], Tanaka [54], and Elliott [55] relate the experimental findings to the structural changes in the first coordination sphere. Other models suggest that changes in the electron energy spectrum are responsible for photoinduced optical phenomena [56]. The photo-induced modifications of the refractive index of thin ChG films are below 0.01, while the absorption coefficient modification is about 10%. Such small modifications raise serious challenges in terms of reproducibility and technology for producing and storing the thin layers. More sensitive experimental methods have to be used for characterizations, as in [57], which presents a study on reversible photo-induced phenomena that occur within amorphous ChG material put in a structure that supports SPR resonance.

In SPR structures, strong changes in the reflected signal can occur even for small changes in the refractive index in amorphous chalcogenide films due to resonance conditions. The authors of [58] demonstrated SPR light modulation using amorphous Ga-La-S films in SPR structures. A prism made of rutile TiO_2_ monocrystal with a high refractive index of refraction was used. As the amorphous As_2_S_3_ has an even higher refractive index (*n* = 2.45), it is difficult to select the right material for prism fabrication. However, the ChG films constitute a planar plasmonic waveguide that can maintain several modes. The effective refractive index *N_eff_* = *β*/*k_0_* of a waveguide depends on the As_2_S_3_ film thickness and can vary from 1 (air refractive index) to 2.45 (amorphous film refractive index). For some thicknesses, the effective refractive index can be lower than the refractive index of BK7 glass, which means that for these thicknesses, the resonant coupling corresponding to the condition *N_eff_* = *n_p_·sinθ* can be realized.

Software in the MATLAB, R2017a application was developed for SPR calculations by the matrix method. Calculations were carried out for a four-layer system. A wavelength of 514 nm was selected to maximize the interaction of light with the amorphous As_2_S_3_ material. This should correspond to the condition αd≈1. We will make the following estimate: for films with a thickness of *d* = *1* μm, the optical absorption coefficient *α* should be about 10^4^ cm^−1^. This corresponds to the selected wavelength. The structure is as follows: BK7(prism)–Au (50 nm metallic film)–As_2_S_3_ (with different film thicknesses)–air. Calculations show that there is only one peak for a small thickness that corresponds to the basic plasmon interaction, while for thicknesses of 250 nm and greater, in addition to the plasmonic dip corresponding to the resonance angle of 65°, there are also peaks corresponding to the guided modes. There are also resonant angles for the incident light, which has s polarization. The numerical simulations indicate that the resonance curves are sharper in the case of coupling with waveguide modes. The reflectivity may change from 0% to 100% only with a modification of the As_2_S_3_ film refractive index of 1%. Such small modifications are known to result in an amorphous As_2_S_3_ film under illumination of the order of 10 mW/cm^2^. The developed model was published in our paper [59]. A detailed calculation of the nonlinear equation shows a hysteresis-type dependence on input power. Here we would like to present some experimental results only.

The experimental schematic was like the one presented in Figure 2b. The four constituent regions have the following refractive indices at a wavelength of 514 nm: a semi-infinite glass of BK7 with the refractive index *n*_1_ = 1.5205, representing the substrate and the coupling prism; a thin metallic layer Au with the complex refractive index [48] *n*_2_ = 0.682–2.020*i*; a thin amorphous As_2_S_3_ film with the complex refractive index *n*_3_ = 2.852–0.019*i*, which was determined from ellipsometry studies; and a semi-infinite air cover region (*n*_4_ = 1). The high refractive index of the amorphous As_2_S_3_ layer forms a plasmonic planar waveguide. There is a sharp drop in the reflection coefficient in the reflectivity of light due to resonant coupling of the incident radiation with waveguide mode when the metallic film thickness is close to 50 nm (according to the calculations).

The amorphous ChG As_2_S_3_ film was obtained by thermal evaporation in a vacuum of 6.6 × 10^−4^ Pa. The technological setup was improved to avoid droplet formation and a high roughness of the film surface. Small granules of As_2_S_3_ obtained from bulk and shredded materials were placed in a tube made of molten quartz. This tube was heated from the outside by a coil made of nichrome, through which current passes. The radiation of the CW argon laser with a beam diameter of 1 mm was directed onto the plasmonic structure through the coupling prism made of BK7 glass. The polarization of the laser is in the plane of incidence, so the TM modes were excited. The power of the incident laser varied in the range of 1 to 20 mW.

**Figure 8:**
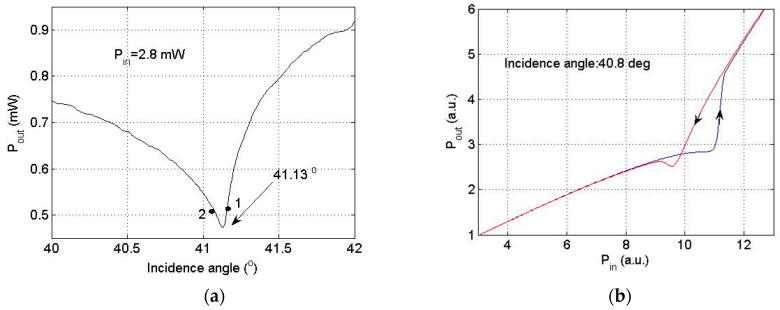
Hysteresis type of the reflected output power in the SPR structure with As_2_S_3_ film. (**a**) Experimental output power recorded at a near-resonance angle of 41.13° at a low incident power of 2.8 mW. (**b**) Output power vs. incident power for an incidence angle of 40.8°. The up arrow on the black curve indicates increasing power, while the down arrow on the red curve indicates decreasing power. Nonlinear hysteresis was established for a laser beam power of 10÷11 mW if the initial angle was adjusted at values lower than the resonance dip (near point 2). Points 1 and 2 in figure (**a**) are indicative, only to differentiate the different behavior of the system for angles higher or lower than the resonance angle equal to 41.13°.

Figure 8 shows the results of the reflected laser power as a function of incident intensity.

The data are very consistent with what the model predicted. At the initial stage, when the power of the incident light was low, the lowest reflection was obtained by setting the angle of incidence. The found angle of incidence was 41.13° (Figure 8a). The angle of incidence was then changed by rotating the structure down to 40.8° (near point 2 on the curve in Figure 8a). In this position, the dependence of the power output *P_out_* as a function of incident power was measured. The results are shown in Figure 8b. A large loop of nonlinear hysteresis was established for laser beam power in the range of 10÷11 mW. Hysteresis was not observed for initial angle settings greater than 41.13°, near point 1.

The reflectance is very sensitive to small changes in the ChG film’s refractive index induced by CW argon laser radiation at 514 nm. In the four-layer configuration, an As_2_S_3_ film with a high refractive index acts as a waveguide. Mode dispersion and self-induced changes of the optical constants lead to an optical hysteresis loop. The shape depends a lot on the initial angle of incidence.

As a conclusion, it can be mentioned that the experiments with amorphous As_2_S_3_ films in SPR configuration established a hysteresis loop in output power. It was demonstrated that the SPR structure with an amorphous As_2_S_3_ film can be a promising medium for active plasmonic devices. Future studies can be carried out in order to obtain optical bistability. The established reflectance depends on laser intensity at a specific wavelength. For many ChG materials used for information recording, the required exposure for inducing optical modifications is of the order of 1÷2 J/cm^2^ [60,61]. Moreover, SPR structures containing a ChG layer that display reversible changes in the photoinduced optical axis enable the engineering of optical memory devices.

## 7. SPR Optical Sensors in a Four-Layer Structure That Contains an Amorphous As_2_S_3_ Film

Optical sensors based on SPR in Kretschmann’s three-layer configuration are an optically pumped and optically interrogated powerful sensing platform, especially for bio-sensing. The main advantage is the real-time measurements permitted to investigate the kinetics of biological reactions. Several instrumental giants like [62,63] entered the market with new high-performance devices after BIACORE, a Swedish company situated in Uppsala [64], was launched as one of the first startups. New startups continue to enter the market [65], which confirms that the commercial base is growing.

A basic feature of a three-layer configuration is its simplicity and high stability because it only uses optical glass and gold film. As far as sensitivity is concerned, this structure has no room for maneuver, with the parameters depending on the material’s optical constants. As shown at the beginning of this article, the four-layer structure has the advanced possibilities to lead with sensitivity (in particular, depth of field), but also as devices with active optical properties. An approach to structure with nonlinear optical properties was examined above. Next, we will unfold the possibilities of the four-layer structure, one of which is an amorphous ChG material film of type As_2_S_3_ for chemical sensors and biosensors.

The operation principle of optical sensors is based on the determination of the change in the refractive index. Optical sensors can determine a wide range of chemical compounds by adjusting the angle of incidence. This is simpler than fiber-optic sensors, which require [8,40,41,66] the scanning of the interrogation wavelength. SPR can be achieved at the interface between a metal film and a dielectric medium. SPR-based devices are known in various configurations [67,68]. Sensors with SPR for hydrogen detection [69], methylene groups [70], hydrocarbons such as ethane and methane [71], nitrogen dioxide [72], etc., are known.

### 7.1. SPR Sensors with As_2_S_3_ for Alcohol Identification

Alcohols (e.g., ethanol) are of great importance since they are used in various areas such as drinks, the food industry, fuel, the environment, security, etc. Alcohols differ in their refractive index, and small changes in the refractive index or the extinction coefficient may be recorded when the created structure is placed in a resonant structure. In some papers [73,74], the optical reflection change for ethanol sensing has been demonstrated in a SPR configuration using thin films of TiO_2_ nanocrystals. The minimum concentration detected amounted to 780 ppm, leading to a 4% change in reflection. The coupling of light with the plasmonic wave was ensured in the Kretschmann configuration [75]. A coupling prism is used to increase the angle of incidence. The resonance conditions are highly dependent on the refractive index of the neighboring medium, so very small changes result in a measurable shift in the resonant dip.

In this section, concrete numerical calculations of SPR for some alcohols of practical importance are presented briefly. The sensor optimization was undertaken by adjusting the thickness of the ChG films. We presented the results in more detail in a previous paper [76]. The structure is in contact with the liquid to be investigated, in our case, alcohol. The specific alcohols are disclosed by measuring the resonance incident angle. Three cases with different thicknesses were considered. The films made of amorphous arsenic sulfide have thicknesses of 800 nm, 1000 nm, and 1100 nm. The wavelength of interrogation was 1550 nm, and the gold film was of 40 nm thickness.

The refractive index of rutile was considered to be between 2.45 (ordinary) and 2.70 (extraordinary). Five alcohols with known refractive indices (methanol, ethanol, propanol, butanol, and pentanol) were considered. Our calculations were performed to establish if alcohols can be distinguished by a four-layer SPR optical device. Using the known alcohol’s refractive index, the resonance angle was calculated. The results are summarized in Table 4.

The ratio Δ*θ*(*R_min_*)/Δ*n* (variation of *θ_min_* with the refractive index) represents the selectivity power to the refractive index. The following parameters were obtained: 4.90°/RIU for *d* = 800 nm; 13.30°/RIU for *d* = 1000 nm; and 8.40°/RIU for *d* = 1100 nm. Note: RIU means refractive index units.

**Figure 9:**
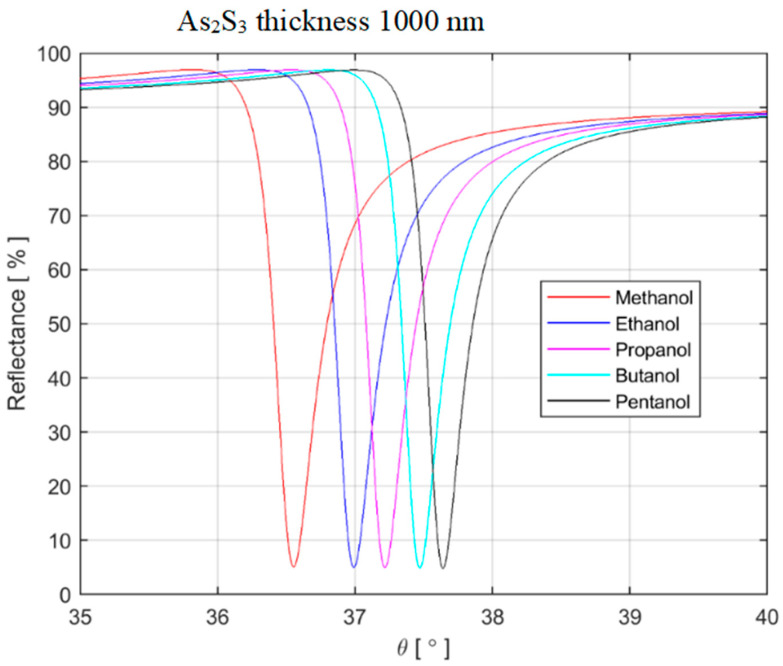
Reflectance of light obtained for several alcohols.

The reflectance curves for a few alcohols are shown in Figure 9. It can be seen that the shift of the dip and the shape of the curve make it possible to clearly identify alcohols.

The four-layer SPR structure is very sensitive to changes in refractive indices. The sensitivity to changes can be modified by adjusting the film’s thickness. The resolution of the refractive index depends on the measured precision of the angle determination and the stability of light. Less than 10^−4^ refractive index changes can be distinguished in our case. The method can be applied not only for alcohol identification but also for the identification of other liquids. The working wavelength of 1550 nm corresponds to fiber-optic information networks, which is an advantage for devices.

### 7.2. SPR Bio-Sensor with an As_2_S_3_ Film for E. coli Bacteria Detection

In the previous section, we presented the calculated characteristics of the four-layer SPR structure for alcohol identification. We published similar analyses for hydrocarbon identification in a previous paper [77]. Further, we will present the use of a four-layer (SPR) structure as a sensor able to detect pathogen strains such as *E. coli* bacteria. More details are presented in our paper [78].

**Figure 10:**
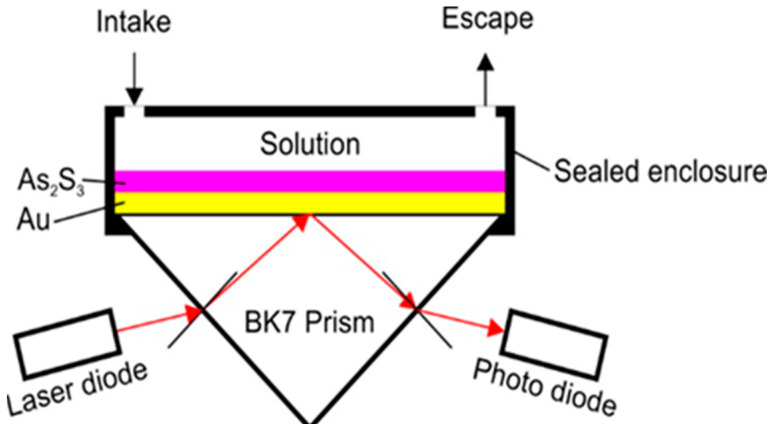
Schematic of the SPR sensor.

In order to avoid working with bacteria that might be unsafe, the aim was to detect the marker—an enzyme produced by the alive bacteria. The marker is named β-galactosidase. The sensor is presented schematically in Figure 10.

It consists of a cell that is pressed onto the plasmonic chipset. The chipset contains gold and amorphous As_2_S_3_ films, usually obtained by vacuum deposition on a glass-plane substrate that is backside-glued to the prism’s base. The liquids for characterization (which can be different chemicals) flow into the cell. The measurements consist of a resonance angle determination. The enzyme was procured from Sigma Aldrich Chemicals Pvt. Ltd., Darmstadt, Germany.

The solution refractive index was found for five concentrations (0%, 0.05%, 0.1%, 0.5%, and 1%) of the solution. The refractive index of the solution was determined as follows: it was considered that the refractive index of the solution changes linearly from that of water *n_w_* (0%) to that of the pure enzyme *n_g_*. Respectively, the following relationship can be written:

(12) $$n_{s}=n_{w}+\left(n_{g}-n_{w}\right)\cdot C[\%]/100$$

Here, unlike in previous situations, in the calculations it was considered that the refractive index of the ambient environment is that of the solution, not that of the air, which has always been considered equal to one. The structure parameters are presented in Table 5.

**Figure 11:**
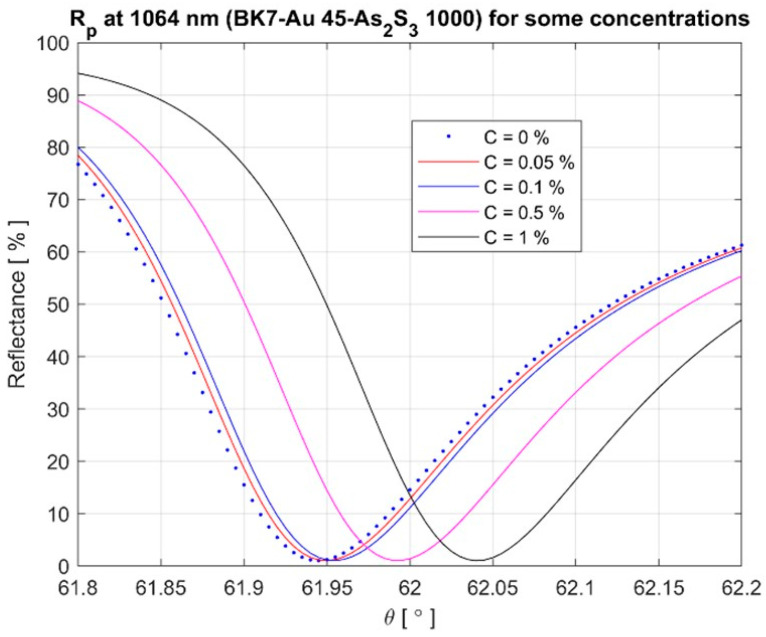
Reflectance vs. incidence angle at different concentrations.

The transfer matrix formalism was used to calculate the reflectance characterizing the plasmonic structure with the parameters presented in Table 5. The results for the reflectance values are presented in Figure 11 for TM polarization. The SPR resonance angle was measured with an accuracy of 1%. The established half-width of the resonance curve was 0.25°.

**Figure 12:**
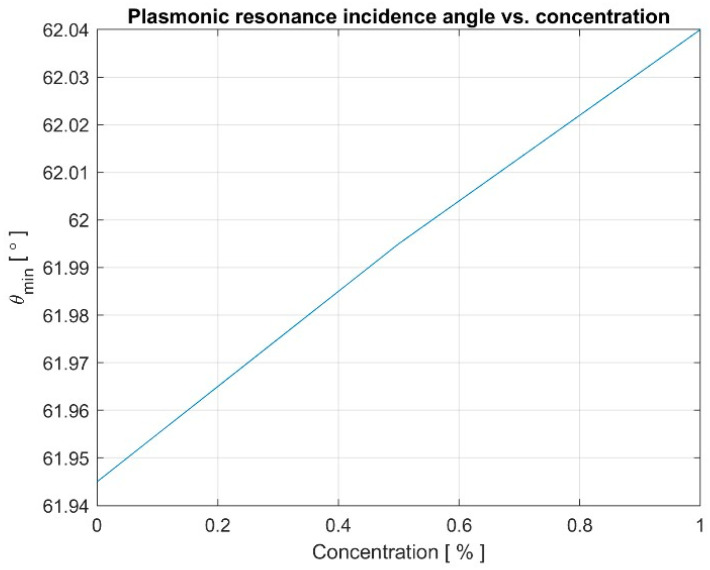
Plasmonic resonance incidence angle vs. solution concentration.

The results presented in Figure 12 show a quasi-linear dependency of the resonance angle on the enzyme concentration.

Research has shown that a waveguide SPR structure made of high-refractive-index materials, like amorphous chalcogenide, has good sensitivity and can be used to identify the presence of *E. coli* bacteria by determining the concentration of a marker enzyme in aqueous solution. This is determined by measuring the angle of resonance. The solution with a certain concentration “resonates” at a specific incidence beam angle, which can be measured.

## 8. Discussion

The SPR concept currently underpins many optical environmental sensors. Several tool platforms are currently traded around the world. Their operation is based on the Kretschmann configuration of coupling light with surface plasmon waves. This interaction is manifested by a very narrow resonance curve, whose position depends on the refractive index of the surrounding environment. In this configuration, which is a three-layer one, the position and width of the resonance curve are fixed and are defined by the optical constants (*n*, *k*) of the metal film and the refractive index of the coupling prism. Of course, there are small changes related to dispersion, but they are insignificant. However, the resonance characteristics can be considerably altered if the SPR structure also includes a film consisting of an optically transparent material. Thus, a four-layer SPR structure is built. The transparent film with a high refractive index can settle on the substrate over the metal film. The opposite side of the substrate is attached to the prism by an immersion oil, which must be made of a material with optical properties close to the substrate. The prism can be used with several chipsets, which wear out after several applications. As a dielectric film, it can serve amorphous ChG materials that also exhibit semiconductor qualities, but for photon energies under the forbidden band, they have good optical transparency.

The four-layer structure acts as a planar waveguide, but the metal film, which is indispensable for achieving the SPR interaction, introduces significant optical losses. Minimizing them is achieved by selecting the thickness, which should be around 50 nm for gold film. The dispersion of gold optical constants is such that for the near-IR spectral domain, thicknesses of 40÷45 nm are more optimal. The calculations are based on the transfer matrix method. The multilayer structure is examined as a multiplication of matrices, so calculations can be made for a structure with an arbitrary number of layers. Calculations have shown that the resonance curves are quite sharp.

The increased refractive index of the film is essential to achieving better field confinement near the surface. In addition to the specific situation related to odd–even modes, the necessary degree of field confinement is achieved by adjusting the positioning of that plasmonic waveguide mode related to the “mode cut-off”. This is ensured by selecting the thickness of the film. Amorphous ChG materials are considered suitable materials for optical waveguide development. Films with the composition As_2_S_3_ and As_2_Se_3_n are considered as reference materials. They have a high refractive index (2.45÷3.00), low optical losses in the transparency band, and can be easily obtained on large metal or dielectric surfaces by vacuum deposition techniques. As can be seen from our reference papers, an optical absorption of 100 cm^−1^ leads to a considerable widening of the resonance contour and, consequently, to a decrease in the device’s performance. Note that 100 cm^−1^ corresponds to optical absorption of only 1% in a film with a thickness of 1 μm.

SPR in a four-layer configuration can always be achieved if a prism with a high refractive index is used, higher than that of the ChG film. The angle at the base of the prism must be chosen to be equal to the calculated incident resonance angle. In this case, the laser beam will be directed normally on the side face of the prism. The exact angle of plasmon resonance was provided with small adjustments to the angle of incidence.

Simulations show that the four-layer SPR configuration allows plasmonic resonance to be obtained for both p polarization (TM mode) and s polarization (TE mode). The coupling prism can be made from GaP, which is an anisotropic material with a refractive index (2.67 and 2.54 in the VIS domain) higher than that of the ChG film with amorphous As_2_S_3_ used as a waveguide. The incident light is coupled with a certain plasmonic waveguide mode through the evanescent field that penetrates the gold film, which is a semi-transparent one with a thickness in the range of 40–50 nm. With such a prism, SPR is obtained in a continuous range of film thicknesses in As_2_S_3_. In terms of incident light polarization, it can be found that *R_p_* minimums are lower and very close to zero compared to *R_s_* minimums. The best gold film thickness required to achieve the clearest resonance in the near-IR band was 40 nm, which differs from the optimal 50 nm thickness known for the visible spectral range. At a wavelength of 1310 nm, an absolute minimum *R_p_* of 0.004% was obtained for a 300 nm As_2_S_3_ layer, while an absolute minimum *R_s_* of 1.43% was obtained for a 500 nm As_2_S_3_ layer. At a wavelength of 1550 nm, the minimums are higher: the minimum *R_p_* is 0.66% for a 1000 nm As_2_S_3_ layer and 1.15% for a 300 nm As_2_S_3_ layer, and the minimum *R_s_* is 5.93% for a 1000 nm As_2_S_3_ layer and 10.72% for a 500 nm As_2_S_3_ layer.

A topical issue is the need to use high-quality, low-cost boro-silicate glass materials (such as BK7) for coupling prisms. It is known that these materials have a refractive index in the range 1.5÷1.7, much lower than the refractive index of ChG materials used to make the plasmonic waveguide. This may seem impossible at first glance. However, films made of ChG materials can withstand planar guide modes with an effective refractive index of less than 1.51, which is the BK7 refractive index in the visible domain. They can be excited by prisms made of material with this refractive index or higher. From the calculations, it was established that SPR can be provided in this case, but only for ChG films, the thickness of which is within a certain range.

Photoinduced changes in optical constants in amorphous ChG are a well-known phenomenon. The value of these changes in ChG thin films is of the order of 10^−2^. Such small changes pose serious challenges in terms of reproducibility and technology. In our work, a model for self-induced nonlinear changes in refractive index in As_2_S_3_ has been developed. The self-induced reflectance change in the SPR resonance structure with As_2_S_3_ films was studied experimentally.

The model has been confirmed experimentally. To begin with, the resonance value for low light intensities was determined, which was 41.13°. Then, a small detuning of the angle from the resonant position on the left branch was made (see point 2 in Figure 8a). A hysteresis loop was obtained by increasing and decreasing the intensity of light (Figure 8b). No hysteresis loop was observed when the initial detuning was at the right branch (see point 1 in Figure 8a) of the resonance curve. Research provided has demonstrated the possibility of realizing bistable optical devices of low intensity in SPR structures with amorphous ChG films.

## 9. Conclusions

Four-layer structures containing amorphous ChG materials open up new possibilities in terms of manipulation with the degree of confinement, sensitivity to refractive index changes, and depth of field. Our research has shown the ability of the SPR structure to identify alcohols or liquid hydrocarbons. The ability of SPR biosensors with amorphous As_2_S_3_ film to detect *E. coli* bacteria has been experimentally demonstrated. The method used was to detect the concentration of the marker, the marker being an enzyme produced by bacteria while alive, namely β-galactosidase. The four-layer configuration with an amorphous As_2_S_3_ film demonstrated the high sensitivity of the method. A 0.05% change in concentration causes an 18 s arcsecond change in the plasmon resonance angle, which can be resolved. The four-layer SPR configuration offers the possibility of developing new nonlinear devices and advanced optical sensors.

Our experiments with amorphous As_2_S_3_ films in the SPR configuration established a hysteresis loop in output power so that they can be promising media for active plasmonic devices. Future studies can be undertaken in order to obtain optical bistability. SPR structures containing a ChG that display reversible changes of the photoinduced optical axis enable the engineering of optical memory devices.

## Figures and Tables

**Table 1:** Optical constants of some usual metals used in SPR experiments.

Metal	Wavelength (μm)	Refractive Index, *n*	Extinction Coefficient, *k*
Au	0.65	0.16	3.15
Au	1.55	0.55	11.5
Ag	0.65	0.14	4.15
Ag	1.55	0.51	10.8
Al	0.65	1.49	7.82
Al	1.55	1.44	16.0

**Table 2:** Minima of reflectance in %. The wavelength is 1310 nm.

Thin Film Thickness, nm	*R_p_*	*R_s_*	*R_p_*	*R_s_*	*R_p_*	*R_s_*
	40 nm Au Film		45 nm Au Film		50 nm Au Film	
As_2_S_3_—300 nm	0.004	3.15	3.32	11.73	12.79	24.06
As_2_S_3_—500 nm	0.02	1.43	3.05	8.30	12.36	19.55
As_2_S_3_—700 nm	0.03	2.88	3.00	11.24	12.28	23.42
As_2_S_3_—1000 nm	0.01	2.25	2.98	10.06	12.26	21.94

**Table 3:** Minima in % for *R_p_* and *R_s_* polarizations at a wavelength of 1550 nm.

ChG Thickness	Au 40 nm		Au 45 nm		Au 50 nm	
	*R_p_*	*R_s_*	*R_p_*	*R_s_*	*R_p_*	*R_s_*
As_2_S_3_, 300 nm	1.15	7.74	8.05	18.58	19.61	31.80
As_2_S_3_, 500 nm	0.77	10.72	7.12	22.51	18.42	36.07
As_2_S_3_, 700 nm	0.69	7.00	6.91	17.53	18.13	30.60
As_2_S_3_, 1000 nm	0.66	5.93	6.82	15.99	18.02	28.87

**Table 4:** Angle of resonance for different alcohols.

Alcohol Type	Refractive Index	*d* = 800 nm	*d* = 800 nm	*d* = 1100 nm
		*θ (R_min_)°*	*θ (R_min_)°*	*θ (R_min_)°*
Methanol	1.317	49.10	36.56	40.18
Ethanol	1.350	49.26	36.99	40.45
Propanol	1.367	49.34	37.22	40.60
Butanol	1.385	49.44	37.47	40.76
Pentanol	1.397	49.50	37.64	40.86

**Table 5:** Parameters of the SPR structure for enzyme identification.

Layer	Material	Refractive Index	Resonance Angle [°]	Thickness, nm
1	BK7	1.5066		-
2	Au	0.3148 + 6.6282i		45
3	As_2_S_3_	2.4693		1000
4	Solution 0%	1.3260	61.94	-
	Solution 0.05%	1.3261	61.95	-
	Solution 0.1%	1.3261	61.95	-
	Solution 0.5%	1.3267	61.99	-
	Solution 1%	1.3274	62.04	-

## Data Availability

The datasets used and/or analyzed during the current study are available from the corresponding author on reasonable request.
